# Novel Technology in the Treatment of Acne Scars: The Matrix-tunable Radiofrequency Technology

**DOI:** 10.4103/0974-2077.69021

**Published:** 2010

**Authors:** M Ramesh, MG Gopal, Sharath Kumar, Ankur Talwar

**Affiliations:** *Department of Dermatology, Kempegowda Institute of Medical Sciences Hospital, Bangalore, India*

**Keywords:** Acne scars, lasers, radiofrequency

## Abstract

**Background:**

Despite the many advances, scarring, particularly acne or pimple scarring, does not have a satisfactory treatment. A new armamentarium in this field is this recently devised matrix-tunable radiofrequency technology, which utilizes radiofrequency emission in the treatment of acne scars.

**Aims:**

To evaluate the efficiency of the new matrix-tunable radiofrequency technology in patients with acne scars of varying sizes.

**Settings and Design:**

A prospective study of 30 randomly selected patients with acne scars was carried out.

**Material and Methods:**

Thirty healthy patients with different types of acne scars – ice pick, box and rolling type – were randomly selected. The scars were either shallow or deep, varied in size from 2 to 20 mm and ranged in number from 10 to 50. These patients were first treated with broad-spectrum antibiotics and local exfoliating agents (topical tretinoin 0.025%) and then subjected to matrix-tunable radiofrequency technology. Each scar was treated at intervals of 1 month. A maximum of four such sittings were carried out. Patients were followed-up every 15 days. Results were noted at the end of 2 months and 6 months. Improvement was assessed by using the visual analog scale (VAS) at 2 months and 6 months, and results were noted in terms of percentage improvement of the whole face by calculating an average of percentage improvement on the basis of interviews of the patient and his/her accompanying relatives. The visual analog scaling was performed by means of high-resolution digital photographs taken at the baseline and at each subsequent visit.

**Results:**

The VAS improvement in scars ranged from 10 to 50% at the end of 2 months to 20 to 70% at the end of 6 months. Of the 30 patients of acne scars, the cosmetic result was excellent (>60% improvement) in four, good (35–60% improvement) in 18 and moderate to poor (<35% improvement) in eight. A few patients reported burning sensation and a mild sunburn-like sensation for about 1 h after treatment. The patients reported a pinkish tone for 2–3 days. Importantly, with the help of some slight make up, all the 30 patients could return to work the following day.

**Conclusion:**

Matrix-tunable radiofrequency technology is a safe and economically viable option for the dermatologists for the treatment of acne scars, because of the effective results coupled with a low downtime.

## INTRODUCTION

Postacne scars are a major cause for disfigurement on the face in young people. Despite the many advances in the past, postacne scarring still does not have a fully satisfactory treatment. Several treatments of the past, such as dermabrasion, microdermabrasion, chemical peeling, laser resurfacing and cosmetic surgery, had disadvantages of either being too mild and ineffective or too aggressive and complicated.

Recently, a new technology, matrix-tunable radiofrequency technology, has become available for the management of acne scars. This recent innovation claims to offer the advantage of being effective even on deep-pitted and box scars. Tunable fractional technology allows the user to emulate any ablative fractional technology, at the same time allowing optimization of treatment depth, area coverage and the ratio of ablation and thermal zone to each patient’s condition, skin type, desired outcome and tolerance of downtime. Thus, it is not wavelength restricted and hence allows the treatment to be individualized according to the specific needs of the patient.

This article discusses the results of a study conducted on 30 Indian patients with varied types of acne scars.

## MATERIALS AND METHODS

Thirty healthy patients (20 females and 10 males, age range 16–35 years) with no history of keloidal tendency and no bleeding tendency were included in this prospective study. The patients included for the study primarily consisted of skin types IV, V and VI. The patients were selected randomly, with all type of scars – ice pick, box and rolling type of scars. An informed consent was taken from the patients prior to their inclusion in the study. A high-resolution digital photograph was taken at the baseline and subsequently at each follow-up. The patients were divided into three categories according to the number of acne scars: mild (10–20 scars), moderate (20–40 scars) and severe (>40 scars).

**Figure 1:**
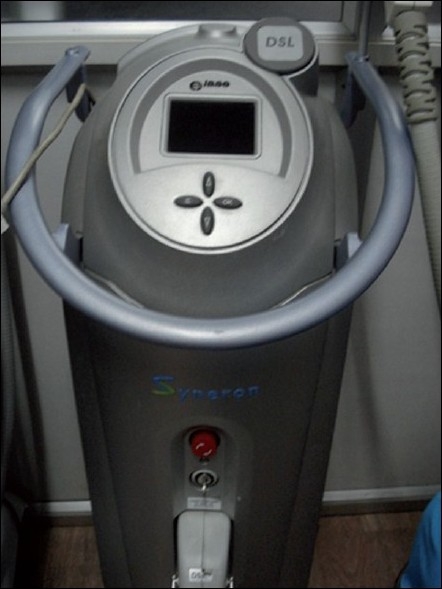
Matrix-tunable radiofrequency machine

**Figure 2:**
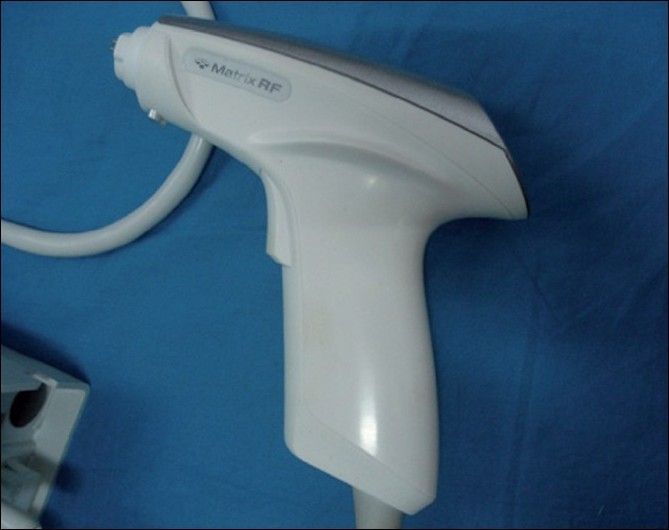
Matrix radiofrequency handset

Patients were first treated with a oral broad-spectrum antibiotic and local exfoliating agents (topical tretinoin 0.025%) and then subjected to matrix-tunable radiofrequency technology (Syneron Corp. Israel.) [Figures 1 and 2]. Eight of the 30 patients were subjected to subcision (for deeper box type scars) prior to the radiofrequency technology while the other 22 were taken up directly.

Each scar was treated at intervals of 1 month. A maximum of four such sittings were carried out. Patients were followed-up every 15 days for possible results as well as for early recognition of side-effects and hence further treatment was not carried out. Results were noted at the end of 2 months and 6 months. All patients were followed-up for a period of 6 months. The dose of radiofrequency varied form 10-20 joules depending on the scar type and the mode of operation(A/B/C).

Improvement was assessed by two independent observers: using visual analog scale (VAS) for the whole face at 2 months and 6 months. The photographs at the baseline were compared with those taken at the end of 2 months and 4 months. The results were noted in terms of percentage improvement by calculating an average of percentage improvement on the basis of interviews of the patient and his/her accompanying relatives. The cosmetic result was graded as excellent (>60% improvement), good (35–60% improvement) and poor (<35% improvement).

## RESULTS

All the patients completed all four sessions of the matrixtunable radiofrequency technology. The treatment was well tolerated with none of the patients reporting any serious side effect requiring discontinuation of therapy. There was obvious clinical improvement in all patients treated, irrespective of the skin types. The VAS improvement in scars ranged from 10 to 50% at the end of 2 months to 20 to 70% at the end of 6 months. The results have been tabulated [Table 1]. Of the 30 patients studied, the cosmetic result was excellent (>60% improvement) in four, good (35–60% improvement) in 18 and moderate to poor (<35% improvement) in eight.

**Table 1:** Visual analog scaling at the end of 2 months and 6 months

Degree of acne scarring	Prior subcision	Predominant scar type	VAS at 2 months (after two sessions of radiofrequency)	VAS at 6 months (after four sessions of radiofrequency)
			
			Showing percentage improvement	
Moderate	No	Rolling	20	40
Mild	No	Ice pick	25	50
Mild	No	Ice pick	30	50
Moderate	No	Rolling	10	30
Moderate	No	Box, rolling	20	50
Mild	Yes	Rolling	50	70
Mild	No	Ice pick, box	40	70
Severe	No	Box	15	30
Mild	No	Ice pick	25	40
Moderate	Yes	Box, rolling	40	60
Moderate	No	Rolling	30	40
Mild	No	Ice pick, box	20	40
Moderate	No	Ice pick	25	45
Severe	Yes	Box, rolling	30	50
Severe	No	Box	10	20
Mild	No	Box	10	30
Moderate	No	Rolling	20	20
Moderate	Yes	Ice pick, rolling	25	60
Moderate	No	Ice pick	35	60
Moderate	Yes	Rolling	40	70
Moderate	Yes	Ice pick, rolling	50	70
Severe	No	Box, rolling	20	30
Mild	No	Rolling	30	40
Moderate	No	Rolling, ice pick	40	40
Moderate	Yes	Rolling	30	50
Severe	Yes	Box	20	40
Moderate	No	Ice pick	25	45
Moderate	No	Box	15	30
Severe	No	Rolling, box	20	30
Mild	No	Ice pick, rolling	25	50

VAS - visual analog scaling

Patients in whom prior subcision had been carried out reported a better outcome as compared to the patients who were recruited directly. Of these 8 patients, the results were excellent (>60% improvement) in three patients and good (35–60% improvement) in the remaining five patients.

**Figure 3:**
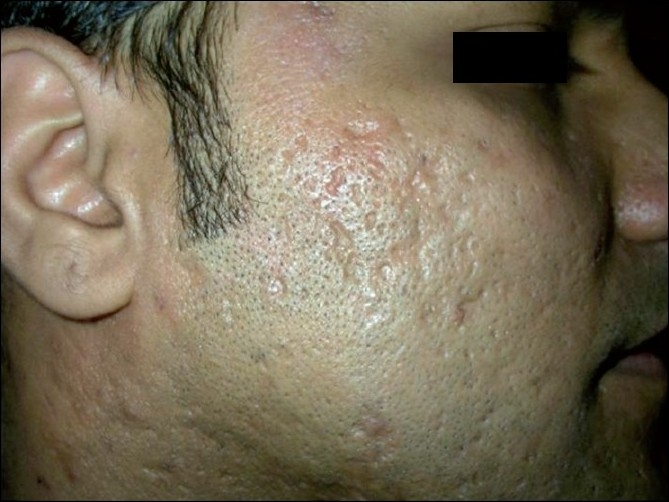
Patient 1, before: showing multiple acne scars on the right cheek

**Figure 4:**
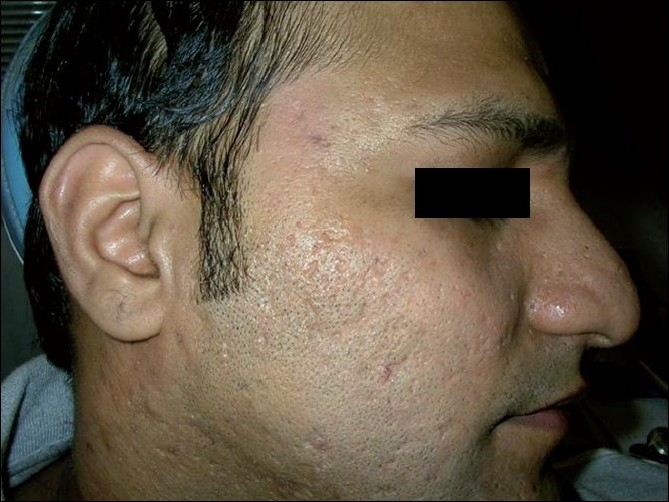
Patient 1, after four sessions of matrix radiofrequency: showing substantial reduction in scars

**Figure 5:**
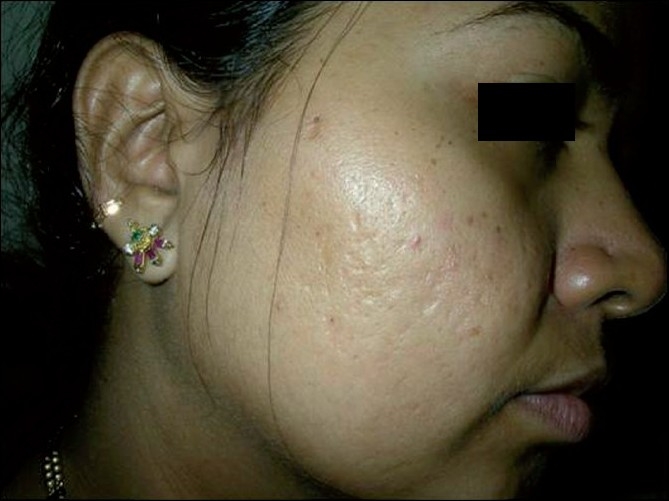
Patient 2, before: picture showing box type and rolling type acne scars on the right cheek

**Figure 6:**
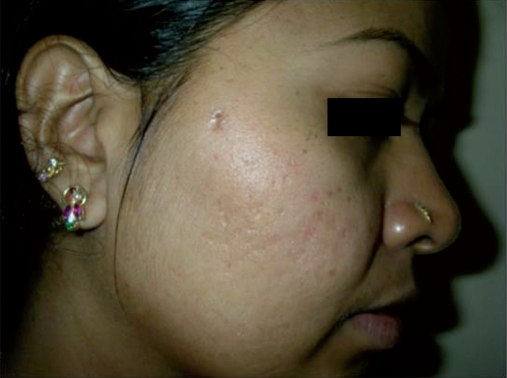
Patient 2, after two sessions of matrix radiofrequency: reduction in the degree of scarring

All the patients with predominant ice pick type of scars (11 patients) reported excellent (20%) to good (80%) results. In contrast, patients with predominant box type of scars (nine patients) showed good results in 45% and moderate to poor results in 55% of the patients, with none of the patients reporting excellent results [Figure 3, 4]. Among patients with predominant rolling scars (10 patients), excellent results were seen in 20% of the patients, good results in 50% of the patients and moderate to poor results in the remaining 20% of the patients [Figure 5, 6].

All patients developed localized treatment-site edema, which generally resolved within 2–3 days. Most patients experienced a burning sensation and a mild sunburn-like sensation for about 1 h and then virtually no discomfort. The skin had a pinkish tone for 2–3 days. Importantly, with the help of some slight camouflage, all the 30 patients could return to work the following day. The patients were advised to use a sunscreen for the next 1 week and some smoothening emollient cream in the night. None of the treated patients developed treatmentsite infection, post-inflammatory pigmentary changes or fibrous nodule.

## DISCUSSION

Acne vulgaris, depending on its severity, can end with a variety of scars. The affected skin of postacne scarring has an abnormal contour, with most scars being depressed below the adjacent normal skin. Many treatment modalities for scar improvement, such as cryoslush with carbon dioxide snow, liquid nitrogen cryopeel, surgical scar revision, electrosurgical planing, chemical peeling, filler substance implantation, dermabrasion, laser abrasion, etc. have been developed. These treatments have the disadvantages of either being too mild and ineffective or of being too aggressive and complicated.

### Review of technology

Matrix-tunable radiofrequency technology is a new modality that has been devised for the treatment of acne scars. Tunable fractional technology allows the user to emulate any ablative fractional technology, depending on the programme selected, thereby optimizing treatment depth, area coverage and the ratio of ablation and thermal zone to each patient’s condition, skin type, desired outcome and tolerance of downtime. Further, another advantage of this technology, which has been claimed, is that it is not chromphore-restricted, i.e. it does not require absorption by any specific chromophore. Thereby, the depth of absorption can be varied depending on the condition being treated. It is the only fractional technology with this unique ability to turn the “heat” on for more collagen production or “off” to safely treat any skin types I – VI, and control the amount of heat delivered, independent of the ablation depth. Matrix RF uses only radiofrequency technology to successfully treat Fitzpatrick skin types IV, V and VI.[1]

With reference to mode of action, it has three components, viz. stimulation of collagenosis, denaturation of collagen and, finally, vaporization[1] The matrix RF applicator is designed to deliver radiofrequency energy to the skin in a nonhomogeneous fractional manner, via an array of multielectrode pins formed of “positively and negatively charged” electrode.[2] The array delivers bipolar RF energy to dry, nonmoisturized skin. This results in heating of areas of the skin that are directly targeted by the multielectrode pins to temperatures leading to ablation and resurfacing of the skin in contact with the multielectrode pins and that directly underneath them. The procedure is carried out while leaving areas of slight or no impact in between the targeted areas that help to maintain the integrity of the treated skin and serve as a reservoir of cells that accelerate and promote the healing process.

During treatment, the multielectrode pin array is placed on the dry skin’s surface. Because the pins are located apart from each other, a different impact is created along the current path not only at varying tissue depths but also in between the pins. The RF current flows between the rows of pins, having the highest impact at electrode–skin contact points, where it creates spots of demarcated ablation[3] and resurfacing of the skin (stratum corneum, the deeper epidermis and superficial dermis).

This technology has got three programmes: A, B and C. Programme A primarily utilizes subnecrotic tissue heating and minimal coagulation of epidermis and dermis as its mode of action (like a Er : Glass laser) and has a very superficial depth of penetration (100–300 μm), with least downtime.[4] Programme B, on the other hand, utilizes minimal ablation and tissue coagulation for its actions and has a moderate depth of penetration (300–500 μm). Thus, it mimics Er: Yag laser in its impact and is indicated for medium depth acne scars and fine wrinkles.[5] Programme C, with a depth of 1,000–1,500 μm causes significant tissue ablation and minimal coagulation and sub necrotic heating. It mimics the CO_2_ laser in its depth and mode of action and is used for deeper acne scars and deep wrinkles[6].

Thus, matrix-tunable radiofrequency technology allows the user to simulate any of the fractional lasers like Er: Yag, Er Glass and CO_2_ by selecting the appropriate programme and thus provides the benefits of three machines in one. However, the major drawback is the disposable tip of the machine, which can be used for a single patient at a time. The tip costs INR 5,000 and thus makes the treatment expensive.

Selecting the appropriate program, coverage and RF level is essential for successful treatment. RF is always started with a low-energy level per program and the skin’s reaction prior to RF is observed before increasing the energy.

The specifications of the RF applicator include:

- RF energy: 2–16 J for 5% coverage and 2–20 J for 10% coverage
- Pulse repetition rate: 1 Hz
- Treatment area footprint: 12 mm × 12 mm

Results appear gradually: immediate transient improvement can be noticed after each treatment. However, maximum long-term effect is usually noticed 2–4 months after treatment conclusion. The number of treatment sessions depends on the individual patient and typically varies between 3 and 4 sessions, every 4–6 weeks. One touch-up session may be needed every 6 months according to individual response.

In our study, the response of patients varied with the type of scars. Patients with ice pick type of scars reported the maximum improvement, while those with predominant box type scars had poor improvement. Rolling type scars had variable results with patients reporting both excellent to poor response to therapy. Performing subcision prior to radiofrequency in box type scars resulted in better improvement. In view of above findings, it can be safely concluded that radiofrequency technology is an effective mode of treatment for ice pick and rolling scars and can also be used for box type scars when combined with prior subcision.

Improper use of the system could result in side-effects. They may appear either at the time of treatment or shortly thereafter. These may include any of following conditions: discomfort or significant pain post-treatment, excessive skin erythema and/or edema, damage to natural skin texture (crust, blister, burn), change of pigmentation (hyper- and hypopigmentation) and scarring.[7] Because the machine has been launched recently (Dec 2008), the results on long-term efficacy have to be yet evaluated. Similarly, there have been very few other clinical trials in assessing its efficacy in patients with acne scarring. In our study, none of the patients reported any serious side effects. The most common adverse effect reported was erythema and burning sensation which subsided on application of topical steroids and sunscreens within 3-5 days. Few patients reported mild scaling and crusting of the facial skin requiring leave from work for 1-2 days.

In conclusion, matrix-tunable radiofrequency technology is a safe option for the dermatologists. The only drawback is the cost of the disposable tip, which may make the treatment expensive and unaffordable for some patients in the Indian setting. Further studies in a larger number of Indian patients is needed to further evaluate the efficacy of this modality.
